# Postoperative Concomitant Chemoradiotherapy Improved Treatment Outcomes of Patients with Oral Cavity Cancer with Multiple-Node Metastases but No Other Major Risk Factors

**DOI:** 10.1371/journal.pone.0086922

**Published:** 2014-02-24

**Authors:** Kang-Hsing Fan, Chien-Yu Lin, Chung-Jan Kang, Li-Yu Lee, Shiang-Fu Huang, Chun-Ta Liao, I-How Chen, Shu-Hang Ng, Hung-Ming Wang, Joseph Tung-Chieh Chang

**Affiliations:** 1 Department of Radiation Oncology, Chang Gung Memorial Hospital, Taoyuan, Taiwan; 2 Department of Hematology and Oncology, Chang Gung Memorial Hospital, Taoyuan, Taiwan; 3 Department of Head and Neck Surgery, Chang Gung Memorial Hospital, Taoyuan, Taiwan; 4 Department of Pathology, Chang Gung Memorial Hospital, Taoyuan, Taiwan; 5 Department of Diagnostic Radiology, Chang Gung Memorial Hospital, Taoyuan, Taiwan; 6 Graduate Institute of Clinical Medical Science, School of Medicine, Chang Gung University, Taoyuan, Taiwan; 7 Department of Medicine, School of Medicine, Chang Gung University, Taoyuan, Taiwan; University of North Carolina School of Medicine, United States of America

## Abstract

**Purpose:**

To investigate the results of postoperative radiotherapy (PORT) for the treatment of pathologic N2b/c squamous cell carcinoma of the oral cavity (OSCC).

**Materials and Methods:**

This study reviewed cancer registry data collected in our hospital from 1998 to 2009 with the following inclusion criteria: primary OSCC, treatment with radical surgery, and multiple nodal metastases. Patients who had extracapsular spreading of the lymph node metastases or positive resection margins or who refused to undergo PORT were excluded. The prescribed dose of PORT was 60–66 Gy. Concurrent chemotherapy was optional. Patient characteristics, treatment parameters and clinical outcome were recorded. The primary end point was overall survival, and the secondary endpoint was disease status.

**Results:**

There were 138 eligible cases, and the median follow-up period was 35 months. The 3-year overall survival rate was 56%. Univariate analysis revealed that pathologic T4 status (pT4), bone marrow invasion, and lymphatic invasion were significantly correlated with poor outcome (*p*<0.05). Multivariate analysis showed that pT4, lymphatic invasion, and the no concurrent chemotherapy were independent poor prognostic factors (*p*<0.05). Fifty-four patients had tumor recurrence. The 3-year recurrence-free survival rate was 59%. Skin invasion, pT4, and bone marrow invasion were correlated with poor prognosis in the univariate analysis (p<0.05). Only pT4 (p<0.01) and no concurrent chemotherapy (p = 0.03) were independently correlated with poor recurrence-free survival.

**Conclusion:**

For OSCC patients with multiple-node metastases without extracapsular spreading or positive resection margins, PORT without concurrent chemotherapy correlated to inferior outcome. Multiple lymph node metastases might be considered an indication for concurrent chemotherapy.

## Introduction

Over the past few years, there have been great advances in the treatment of advanced squamous cell carcinoma of the oral cavity (OSCC) and the management of advanced disease. The principles of treatment are based on the results of randomized trials for all head and neck cancers. Nodal metastasis with extracapsular spreading and positive surgical margins are the strongest risk factors, and these risk factors are clear indications for postoperative radiotherapy (PORT) with concurrent chemotherapy. [1], [2] The influences of other pathologic tumor characteristics on outcomes are still debatable. The involvement of 2 or more regional lymph nodes is one of the controversial issues. The involvement of 2 or more regional lymph nodes was an inclusion criterion for one randomized trial [2], but a combined analysis of that trial and another large postoperative concurrent chemoradiation (CCRT) trial did not suggest that this factor was an indication for CCRT. [3] However, several retrospective analyses revealed a correlation between multiple-node metastases and a higher risk of tumor recurrence. Greengerg and colleagues showed that pathologic stage N2 was correlated with poor prognosis in patients with tongue cancer, regardless of the status of extracapsular spreading. [4], [5] Several analyses of the treatment of oral cavity cancer have also shown that patients with multiple-node metastases have a higher risk of tumor recurrence. [6]–[9] Therefore, excluding these patients from postoperative CCRT is worrisome. When exploring the details of these studies, we found many differences between them. The studies recruited patients with different diseases (all head and neck cancers or OSCC only) or did not narrow down the subject pool on the basis of disease status (multiple-node metastases with or without extracapsular spreading)., We therefore performed a retrospective study to determine the effectiveness of postoperative chemoradiation for the treatment of OSCC in patients with multiple-node metastases.

## Materials and Methods

With the permission of the institutional review board of the hospital, we retrieved clinical data for OSCC patients with pathologic N2b/c stage (American Joint Committee on Cancer (AJCC) staging system, 7th edition) [10] cancer from the cancer registry of our hospital. After further exclusion, 154 patients were selected for analysis from about 5000 OSCC patients. Exclusion criteria included single-node metastasis, the presence of (or no information regarding) positive resection margins, the presence of (or no information regarding) extracapsular spreading in metastatic nodes, a history of previous cancer, a second synchronous cancer, and no standard neck dissection (at least supraomohyoid dissection). A review of the treatment records revealed that 16 patients did not receive adjuvant radiotherapy, and they were excluded from the analysis. The remaining 138 patients completed radical surgery and the entire course of PORT in our department between 1998 and 2008. All data were reviewed after retrieval. Tumor staging was based on the pathology findings and revised according to the 2007 version of the AJCC staging system for analysis. In addition, we recorded the status of the surgical margins, the degree of histologic differentiation, the site of origin, laterality, tumor size, perineural invasion, lymphatic permeation, skin invasion, bone invasion, and invasion depth. Because this study focused on the relationship between nodal status and clinical outcome, we also recorded the presence of low neck (level IV/V) nodal metastases.

All patients received postoperative radiotherapy consisting of a conventional fractionated dose of 1.8 or 2 Gy at 1 fraction per day, 5 days per week using a 6-MV photon beam, for a total dose of 60 to 66 Gy. The initial treatment volume included the primary tumor bed with general margins and the regional cervical lymph nodes. Before 2001, most treatments used the conventional field arrangement, including a bilateral opposing field and a low anterior portal. The spinal cord was shielded after 46 to 46.8 Gy was given. The tumor bed was boosted by the coning down method and by treatment with 6-MV X-rays. The posterior and lower cervical lymph nodes were boosted using an electron beam, if necessary. After 2001, 3D conformal techniques (3DCRT) and intensity modulations (IMRT) were widely applied. Using 3DCRT or IMRT, the maximal dose delivered to the spinal cord and brain stem was limited to 50 Gy. This dose constraint was the first priority in the RT planning. Without violation of this constraint, 95% of the clinical tumor volume and 90% of the planning treatment volume should be irradiated to 100% of the prescribed dose, and the below-dose area should be limited to the surface of the skin or the air cavity in the body. The initial treatment volume included the tumor bed and the regional lymphatics. After the administration of 46 to 50 Gy, the treatment area was reduced to irradiate only the tumor bed and regions with metastatic nodes.

Concurrent chemotherapy was based on cisplatin and administered either low or high dose. For low dose cisplatin, the prescribed dose of cisplatin was 40–50 mg/m^2^ weekly or biweekly, with or without additional oral 5-fluorouracil prodrug. [11], [12] For high dose cisplatin, the prescribed dose of cisplatin was 100 mg/m^2^ triweekly. Chemotherapy was paused or reduced in dose if there were obvious complications.

Outcome measures included local recurrence, regional recurrence, distant metastasis, second primary cancer, and death. All patients were followed at outpatient department every 3–4 months in first 3 years after treatment and 6–12 months after then. Image studies including computer tomography, magnetic resonance image, or ultrasonography were performed annually or when signs of recurrence observed. The re-staging study in patients with a recurrent tumor or a second primary cancer was used to define the tumor extension. Salvage treatment or the best supportive care was given depending on the status of the disease and the patient. If there was any evidence of tumor recurrence, the cause of death was reviewed in detail to determine the failure pattern. The cause of death was recorded as “disease” until every other suspicion was ruled out. Recurrence was verified by pathological examination or consequent clinical findings if no tissue was available. Once recurrence was verified, the date was calculated starting from the day of the first note in the chart indicating signs of possible recurrence. Second primary cancers, death unrelated to recurrence or complications were not counted as treatment failure. The primary end point was overall survival (OS), and the secondary end points were recurrence-free survival (RFS) and local-regional recurrence-free survival (LRRFS). The period of survival was computed from the date of radical surgery to the date of the event; the event was tumor recurrence or “death from disease” for RFS and death for overall survival. All characteristics and treatment parameters were categorized according to available references. Difference of characteristics and treatment parameter between CCRT and RT group were assessed with chi-squared test or Fisher’s exact test (if the sample size was less than 5). We used the Kaplan-Meier method for survival analysis and the log-rank test to determine whether there were significant differences between the patients in terms of the end points. The significance of each survival difference was determined with the log-rank test. Multivariate analysis was performed using Cox regression model to assess the ability of prognostic factors to predict survival outcomes. (expressed as the odds ratio and 95% CI). The correlations of each variable (age, sex, habits regarding alcohol/betel nut/smoking, margin status, overall stage, T stage, N stage, number of nodal metastases, low neck node, invasion depth, perineural invasion, skin invasion, bone invasion, lymphatic invasion, vascular invasion, histologic differentiation, radiation dose less than 66 Gy, concurrent chemotherapy, and the regimen of chemotherapy) to the end points were evaluated by both univariate and multivariate analyses. Differences were considered significant when the p value was less than 0.05. We used the commercial statistics package SPSS 11.0 (SPSS Inc., Chicago IL).

## Results

### Patient Population

Among the 138 patients, the age ranged from 29 to 75 years old, with a median of 49 years. One hundred twenty-seven (90.9%) patients were male, and 11 (9.1%) were female. The most common subsite was the tongue (64, 46.4%), followed by the buccal mucosa (39, 28.3%), gums (13, 9.1%), retromolar trigone (10, 7.2%), mouth floor (5, 3.6%), hard palate (4, 2.9%), and lips (3, 2.2%). All patients had pathologic stage IVa disease, and their nodal stage was either N2b (124, 89.9%) or N2c (14, 10.1%). There were 16 (13.6%), 65 (46.8%), 14 (9.7%), and 43 (29.8%) patients with pathologic stage T1, T2, T3, and T4 disease, respectively. Other characteristics are listed in Table 1.

**Table 1:** Characteristics of all patients.

Characteristic	Subcategory	Frequency (%)	Frequency in the CCRT group (%)	Frequency in theRT group (%)	Exact Significance(2-sided)
Sex	Male	127 (92%)	75 (97.4%)	52 (85.2%)	0.01^a^
	Female	11 (8%)	2 (2.6%)	9 (14.8%)	
Age (Median: 49 (29–75))	<40 years	24 (17.4%)	14 (18.2%)	10 (16.4%)	0.78
	≧40 years	114 (82.6%)	63 (81.8%)	51 (83.6%)	
Smoking	Yes	120 (87%)	69 (89.6%)	51 (83.6%)	0.3
	No	18 (13%)	8 (10.4%)	10 (16.4%)	
Alcohol	Yes	107 (77.5%)	62 (80.5%)	45 (73.8%)	0.35
	No	31 (22.5%)	15 (19.5%)	16 (26.2%)	
Betel quid	Yes	104 (75.4%)	61 (79.2%)	43 (70.5%)	0.24
	No	34 (24.6%)	16 (20.8%)	18 (29.5%)	
Site	Tongue	64 (46.4%)	30 (39%)	34 (55.8%)	0.03^a^
	Mouth floor	5 (3.6%)	1 (1.3%)	4 (6.6%)	
	Lips	3 (2.2%)	2 (2.6%)	1 (1.6%)	
	Buccal mucosa	39 (28.3%)	21 (27.3%)	18 (29.5%)	
	Gums	13 (9.4%)	10 (13%)	3 (4.9%)	
	Hard palate	4 (2.9%)	3 (3.8%)	1 (1.6%)	
	Retromolar trigone	10 (7.2%)	10 (13%)	0	
Differentiation	Well	26 (18.8%)	9 (11.7%)	17 (27.9%)	0.02^a^
	Moderate	90 (65.2%)	52 (67.5%)	38 (62.3%)	
	Poor	22 (16%)	16 (20.8%)	6 (9.8%)	
Pathologic T stage	T1	16 (11.6%)	4 (5.1%)	12 (19.7%)	<0.01^a^
	T2	65 (47.1%)	32 (41.6%)	33 (54.1%)	
	T3	14 (10.1%)	11 (14.3%)	3 (4.9%)	
	T4	43 (30.2%)	30 (39%)	13 (21.3%)	
N stage	N2b	124 (89.9%)	71 (92.2%)	53 (86.9%)	0.3
	N2c	14 (10.1%)	6 (7.8%)	8 (13.1%)	
Margin distance	<5 mm	39 (28.3%)	25 (32.5%)	14 (23%)	0.22
	≧5 mm	99 (71.7%)	52 (67.5%)	47 (77%)	
Low neck lymph node	Yes	6 (4.3%)	3 (3.9%)	3 (4.9%)	1
	No	132 (95.7%)	74 (96.1%)	58 (95.1%)	
Skin invasion	Yes	9 (6.5%)	5 (6.5%)	4 (6.6%)	1
	No	129 (93.5%)	72 (93.5%)	57 (93.4%)	
Bone invasion	Yes	25 (18.1%)	19 (24.7%)	6(9.8%)	0.25
	No	113 (81.9%)	58 (75.3%)	55 (90.2%)	
Perineural invasion	Yes	54 (39.1%)	39 (50.6%)	15 (24.6%)	<0.01^a^
	No	84 (60.9%)	38 (49.4%)	46 (75.4%)	
Vascular invasion	Yes	2 (1.4%)	2 (2.6%)	0	0.21
	No	136 (98.6%)	75 (97.4%)	61 (100%)	
Lymphatic invasion	Yes	16 (11.6%)	11 (14.3%)	5 (8.2%)	0.27
	No	122 (88.4%)	66 (85.7%)	56 (91.8%)	
Invasion depth of tumor	<10 mm	47 (34.1%)	27 (35.1%)	20 (32.8%)	0.78
	≧10 mm	91 (65.9%)	50 (64.9%)	41 (67.2%)	

a Significant difference, *p* – value <0.05.

### Radiotherapy and Chemotherapy

After radical surgery, 91 (65.9%) patients started PORT within 6 weeks, and 26.1% started within 8 weeks. Sixty-nine (50%) patients received PORT with the conventional technique. The other patients received PORT based on 3-dimensional (32, 23.2%) or intensity-modulated radiotherapy (37, 26.8%). Eight patients did not complete the entire radiotherapy course. Among these 8 patients, 5 received PORT without concurrent chemotherapy. Three patients died from acute adverse events. CCRT did not correlate with incomplete radiotherapy (Fisher’s exact test, p = 0.238, one-sided). Eighty-two (58.7%) patients received a total dose of PORT of 66 Gy, and 48 (41.3%) patients received a total dose between 60 and 64.8 Gy. Therefore, the median radiation dose was 66 Gy. PORT was completed within 8 weeks for 109 (79%) patients, and 29 patients (21%) did not because of intolerable adverse events. In total, 66 (47.8%) patients and 11 (8%) patients received weekly and tri-weekly cisplatin-based chemotherapy, respectively. All patients who planned to receive tri-weekly chemotherapy completed at least 2 cycles of chemotherapy. Fifty-four (81.8%) patients in the weekly chemotherapy group received 4 cycles of chemotherapy or more. The details of the treatment-related variables are listed in Table 2. There were significant differences between the 2 treatment groups. In general, the CCRT group had more male patients, more cancers of gums and the retromolar trigone, and more advanced disease features. In addition, the radiation dose was higher and IMRT was more common in the CCRT group. However, CCRT did not delay the initiation or prolong the duration of PORT (tables 1 and 2).

**Table 2:** Treatment parameters of all patients.

Characteristic		Frequency (%)	Frequency in theCCRT group (%)	Frequency in theRT group (%)	Significance of difference
RT technique	2D	69 (50%)	21 (27.2%)	48 (78.7%)	<0.01^a^
	3D CRT	32 (23.2%)	27 (35.1%)	5 (8.2%)	
	IMRT	37 (26.8%)	29 (37.7%)	8 (13.1%)	
RT duration (Median: 49 days(41–81) )))	Incomplete RT	8 (5.8%)	3(3.9%)	5 (8.2%)	0.52
	≦8 weeks	101 (73.2%)	14 (18.2%)	37 (60.8%)	
	>8 weeks	29 (21%)	60 (77.9%)	14 (23%)	
Time between OP & RT	≦6 weeks	91 (65.9%)	49 (63.6%)	42 (68.8%)	0.52
	>6 weeks	47 (34.1%)	28 (36.4%)	19 (31.2%)	
RT dose (Median: 6600 cGy)	Incomplete RT	8 (5.8%)	3(3.9%)	5 (8.2%)	<0.01^a^
	6000–6600 cGy	48 (34.8%)	14 (18.2%)	35 (57.4%)	
	6600 cGy	82 (59.4%)	60 (77.9%)	21 (34.4%)	
Chemotherapy	No chemotherapy	61 (44.2%)	0	61 (100%)	NA
	Low dose cisplatin	66 (47.8%)	66 (85.7%)	0	
	High dose cisplatin	11 (8%)	11 (14.3%)	0	
Total cisplatin dose	No chemotherapy	61 (44.2%)	0	61 (100%)	NA
	Less than 200 mg/m^2^	26 (18.8%)	26 (33.8%)	0	
	200 mg/m^2^ or more	51 (37%)	51 (66.2%)	0	

a Significant difference, *p* – value <0.05.

Abbreviation: NA – not available.

### Overall Survival

**Figure 1:**
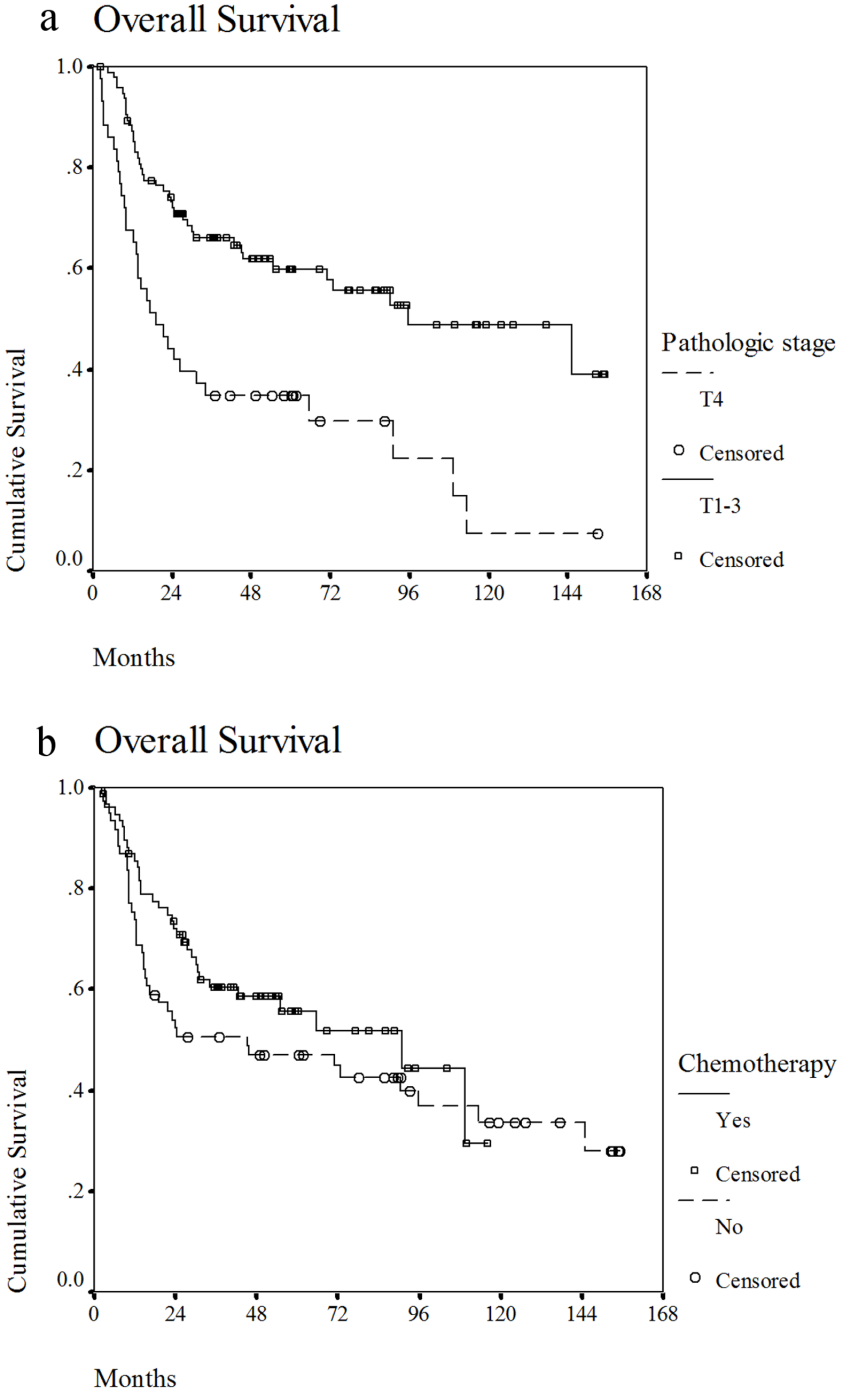
Overall survival curve for patients with pathologic T4 disease and patients treated with concurrent chemotherapy. a. Overall survival curve for patients with pathologic T4 or T1-3 disease. b. Overall survival curve for patients treated with or without concurrent chemotherapy.

At the time of the analysis, 72 patients had died, and 64 patients were alive. The cause of death was cancer recurrence in 47 cases, treatment-related adverse events in 6 cases, second primary cancer in 12 cases, other disease in 5 cases, and unknown cause in 2 cases. The 3-year OS rate of all patients was 56%, and the median survival was 35 months. In the univariate analysis, we found that pathologic T4 disease, bone invasion and lymphatic invasion were correlated with poor OS (*p*<0.05) (Table 3). In the multivariate analysis, pathologic T4 disease (*p*<0.01), no chemotherapy (*p* = 0.05) and lymphatic invasion (*p*<0.01) were statistically significant poor prognostic factors (Table 4). Other treatment parameters, such as the RT technique and dose, were not correlated with the different outcomes. Figure 1 shows the overall survival curves for patients with pathologic T4 disease or disease of another stage and for patients treated with or without concurrent chemotherapy.

**Table 3:** Outcome by patient characteristic and treatment parameter.

Characteristic	Subcategory	3-year OS (*p* value)	3-year RFS (*p* value)	3-year LRFS (*p* value)
Sex	Male	56% (0.81)	59% (0.94)	68% (0.46)
	Female	56%	56%	56%
Age	<40 years	63% (0.32)	66% (0.27)	70% (0.57)
	≧40 years	55%	57%	67%
Smoking	Yes	59% (0.27)	60% (0.66)	69% (0.17)
	No	39%	53%	53%
Alcohol	Yes	57% (0.99)	58% (0.44)	67% (0.65)
	No	55%	64%	70%
Betel quid	Yes	55% (0.28)	58% (0.54)	68% (0.99)
	No	59%	61%	65%
Differentiation	Poor	58% (0.98)	65% (0.58)	83% (0.16)
	Well or Moderate	56%	58%	65%
pT stage	pT4	35% (<0.01)^a^^,^ b	36% (<0.01)^a^^,^ b	49% (<0.01)^a^^,^ b
	pT1-3	66%	69%	74%
pN stage	N2b	59% (0.15)	61% (0.14)	69% (0.41)
	N2c	36%	36%	50%
Surgical margin	≧5 mm	59% (0.07)	61% (0.27)	69% (0.41)
	<5 mm	48%	52%	50%
Skin invasion	Yes	44% (0.58)	22% (0.02)^a^	31% (0.05)^a^
	No	57%	62%	70%
Bone invasion	Yes	32% (<0.01)^a^	41% (0.01)^a^^,^ b	51% (0.01)^a^
	No	62%	63%	71%
Perineural invasion	Yes	59% (0.7)	58% (0.88)	64% (0.52)
	No	55%	60%	70%
Vascular invasion	Yes	100% (0.59)	50% (0.28)	50% (0.12)
	No	55%	59%	68%
Lymphatic invasion	Yes	31% (0.03)^a^^,^ b	53% (0.23)	61% (0.28)
	No	60%	60%	68%
Invasion depth	≧10 mm	50% (0.13)	57% (0.2)	69% (0.95)
	<10 mm	68%	63%	65%
Low neck lymph node	Yes	67% (0.41)	67% (0.68)	83% (0.42)
	No	56%	58%	67%
RT technique	2D	51% (0.44)	54% (0.21)	64% (0.24)
	3D or IMRT	61%	63%	71%
RT duration	≦8 weeks	56% (0.34)	56% (0.17)	66% (0.41)
	>8 weeks	65%	71%	74%
Time between OP & RT	≦6 weeks	56% (0.97)	57% (0.7)	66% (0.88)
	>6 weeks	57%	62%	70%
RT Dose	<6600 cGy	48% (0.3)	58% (0.66)	70% (0.97)
	6600 cGy	61%	59%	65%
Chemotherapy	No	51% (0.21)	52% (0.25)	63% (0.71)
	Yes	60%	65%	70%
Chemotherapy regimen	Low dose	60% (0.4)	61% (0.3)	66% (0.15)
	High dose	70%	82%	91%
Total cisplatin dose	<200 mg/m^2^	51% (0.11)	52% (0.04)^a^^,^ b	63% (0.245)
	≧200 mg/m^2^	65%	70%	74%

a statistically significant in the univariate analysis, p<0.05.

b statistically significant in the multivariate analysis, p<0.05.

**Table 4:** Significant variables in the multivariate analyses for overall survival and recurrence-free survival.

	OS *p* - value	OS HR (95% CI)	RFS *p* - value	RFS HR (95% CI)	LRFS *p* - value	LRFS HR (95% CI)
Pathologic T4 stage	<0.01	2.86 (1.72–4.76)	<0.01	3.22 (1.85–5.59)	<0.01	2.63 (1.37–5.04 )
Lymphatic invasion	0.01	2.877 (1.45–5.7)	NS	NS	NS	NS
No chemotherapy	0.05	1.683 (1.01–2.82)	0.03	1.84 (1.06–3.19)	NS	NS

Abbreviation: NS – not significant.

### Recurrence-free Survival and Local-regional Recurrence-free Survival

**Figure 2:**
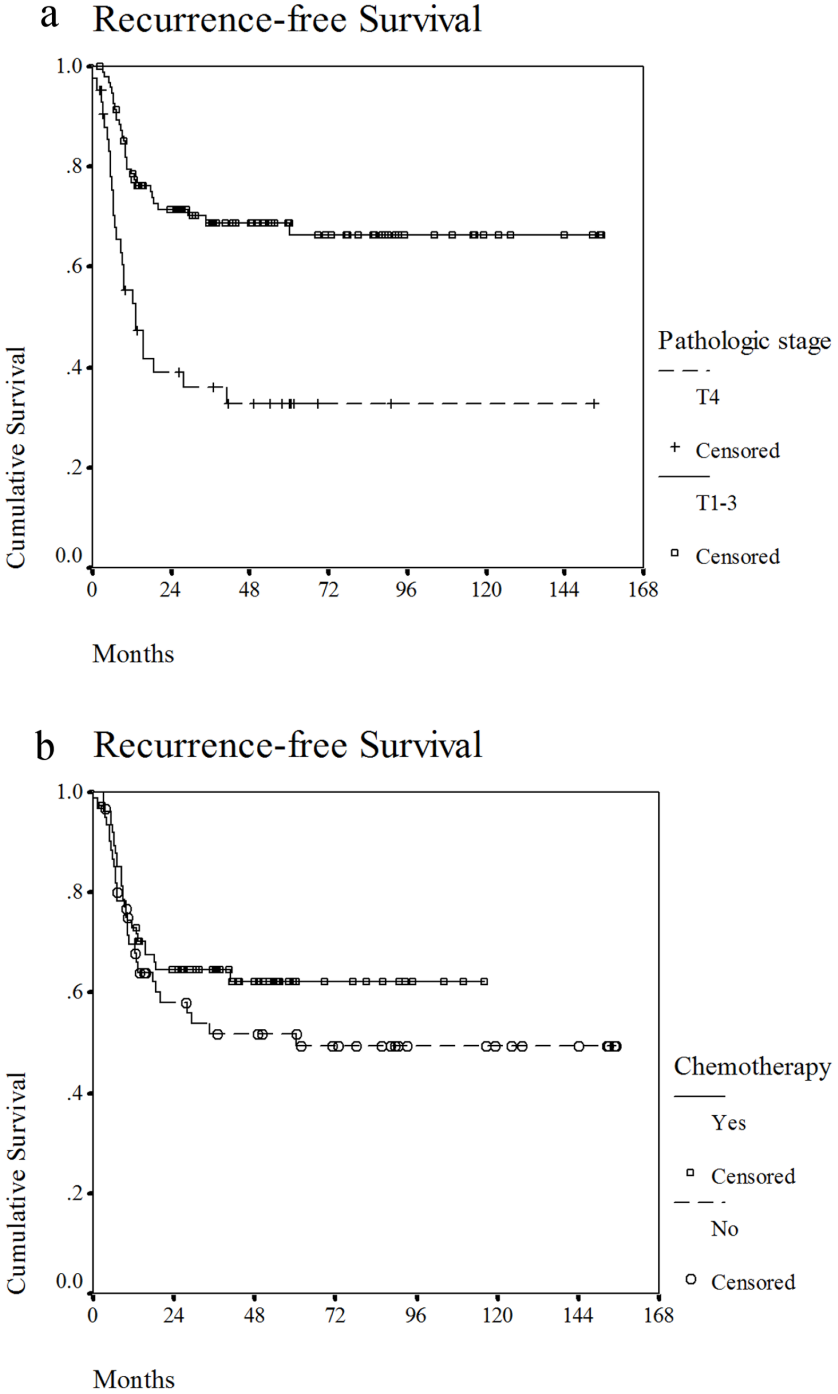
Recurrence-free survival curve for patients with pathologic T4 disease and patients treated with concurrent chemotherapy. a. Recurrence-free survival curve for patients with pathologic T4 or T1-3 disease. b. Recurrence-free survival curve for patients treated with or without concurrent chemotherapy.

Fifty-two patients had documented disease recurrence. Two patients who died from unknown causes were also counted as experiencing disease recurrence. One patient had tumor recurrence diagnosed at another hospital, but there was no definite information on the location. The 3-year RFS rate for all patients was 59%. Nodal recurrence (18) was the most common first recurrence pattern, followed by local (15), distant (11), and both local and nodal recurrences (8). Of the 53 patients who had definite tumor recurrence, only 6 were salvaged by surgery and/or radiotherapy. In the univariate analysis, we found that pathologic T4 disease, bone invasion, and skin invasion were significantly correlated with poor RFS (*p*<0.05) (Table 3). In the multivariate analysis, pathologic T4 disease (p<0.01) and no concurrent chemotherapy (p = 0.03) were independently correlated with tumor recurrence (Table 4). Figure 2 shows the recurrence-free survival curves for patients with pathologic T4 disease or disease of another stage and for patients treated with or without concurrent chemotherapy. Forty-one patients developed local-regional recurrences. The 3-year LRRFS was 67%. Pathologic T4 disease, skin invasion, and bone invasion were significantly correlated with poor LRRFS (*p*<0.05) (Table 3). However, only pathologic T4 disease was an independent poor prognostic factor for LRRFS (*p*<0.01) (Table 4).

The effect of CCRT was further tested. The differences of OS, RFS, and LRFS with or without CCRT were further tested with stratification by pathologic T stage, lymphatic invasion, presence of other pathologic risk factors, and cumulative dose of cisplatin. The difference reached significance only for the OS (*p* = 0.04) of patients with pT1-3 disease and cumulative cisplatin dose ≧200 mg/m^2^ the RFS. (Table 5) The 3-year RFS was 52% and 70% when cumulative cisplatin <200 mg/m^2^ or ≧200 mg/m^2^ (p = 0.04). The 3-year RFS was 54% and 52% in patients who did not receive chemotherapy and chemotherapy was given with cumulative cisplatin dose <200 mg/m^2^ (p = 0.46). In multivariate analysis, cumulative cisplatin <200 mg/m^2^ (p = 0.03) replaced the role of CCRT and became the independent poor prognostic factor with pathologic T4 disease (p<0.01). To avoid the effect of interaction between incomplete radiotherapy and cumulative cisplatin dose <200 mg/m^2^, analyses of RFS were done again after removing patients who did not complete radiotherapy. The result was similar. The 3-year RFS were 54% and 69% in patients received total cisplatin less than 200 mg/m^2^ and equal to or more than 200 mg/m^2^ (p = 0.05). The 3-year RFS was 54% and 52% in patients who did not receive chemotherapy and chemotherapy was given with cumulative cisplatin dose <200 mg/m^2^ (p = 0.67). And cumulative cisplatin dose less than 200 mg/m^2^ was an independent poor prognostic factor in multivariate analysis (p = 0.02, hazard ratio = 2.081, 95% confidence interval: 1.106–3.915).

**Table 5:** OS and RFS for patients treated with or without CCRT stratified based on different factors.

		3-year OS	*p* – value	3-year RFS	*p* – value	3-year LRFS	*p* – value
pT4	CCRT	40%	0.44	43%	0.36	53%	0.9
	No CCRT	23%		20%		34%	
pT1-3	CCRT	65%	0.04	78%	0.06	80%	0.25
	No CCRT	44%		56%		69%	
Lymphatic invasion	CCRT	36%	0.40	60%	0.52	67%	0.72
	No CCRT	20%		40%		53%	
No lymphatic invasion	CCRT	65%	0.16	65%	0.27	72%	0.71
	No CCRT	47%		54%		65%	
No other risk factors	CCRT	50%	0.69	67%	0.85	67%	0.85
	No CCRT	50%		60%		60%	
With other risk factor	CCRT	61%	0.25	64%	0.23	83%	0.44
	No CCRT	51%		51%		70%	
Cumulative cisplatin dose	≧200 mg/m^2^	65%	0.11	70%	0.04	74%	0.25
	<200 mg/m^2^	51%		52%		63%	

### Lethal Adverse Events, Second Primary Cancers, and other Death Events

Three patients died from acute adverse events during PORT (2.2%). Two of them were undergoing concurrent chemotherapy and died from infection. The other patient received radiotherapy alone and died from aspiration pneumonia. Three patients (2.2%) died from aspiration pneumonia more than 3 months after treatment completion, and these deaths were classified as lethal chronic adverse events. CCRT did not correlate with lethal toxicity neither during PORT (Fisher’s exact test, p = 0.587, one-sided) nor all time (Fisher’s exact test, p = 0.237, one-sided). Twenty-two patients developed second primary cancers during the follow-up period. Head and neck cancers were the most common type and occurred in 13 patients. There were also 4 lung cancers, one leukemia, one upper urinary tract cancer, one esophageal cancer, one gastric cancer, and one skin cancer during the follow-up period. Three patients had more than 3 primary cancers, and all of these cancers were in the head and neck region. Five patients died from various causes other than cancer, including cardiovascular disease, cerebrovascular accidents, and renal failure.

## Discussion

For many years, physicians have sought to determine the “right” treatment for cancer patients. Toxic treatment should be avoided if possible. Postoperative radiotherapy has been incorporated into the treatment of head and neck cancers for years. Recent clinical trials have confirmed the role of postoperative CCRT in the treatment of head and neck cancers with positive resection margins or lymph node metastases with extracapsular spreading. [1], [2] However, as mentioned above, whether the presence of multiple-node metastases is an indication for CCRT is still controversial. According to our previous studies, the presence of multiple-node metastases was correlated with higher tumor recurrence and poor overall survival. [9], [13] Therefore, the presence of multiple-node metastases is still an indication for postoperative CCRT for the treatment of head and neck cancers in some hospital. This study confirmed that this decision is appropriate. Postoperative CCRT is likely to reduce the recurrence risk of OSCC patients with multiple-node metastases, especially when total dose of cisplatin reached 200 mg/m^2^.

Although CCRT was correlated with better treatment outcomes in the multivariate analysis, the differences in the univariate analysis were not significant in each category, as presented above. This lack of significance was caused by the unbalanced distribution of the patient groups. As shown in table 1 and table 2, the patients in the CCRT group had more advanced OSCC. Thus, adjuvant CCRT could reduce the risk of recurrence and result in a similar or better outcome. Table 5 shows the results of the analysis with stratification. The survivals were improved by CCRT around 10% to 20% in each categories except patients who had no other pathologic risk factors. The difference was not significant since the sample size in each categories were too small after stratification. But at least significant benefits of CCRT were shown in patients with pathologic T1-3 disease and when cumulative cisplatin dose was equal to or more than 200 mg/m^2^. A constantly occurred by insignificant benefit and statistically significant result in 2 stratification methods can also explained the independent prognostic power of CCRT in multivariate analysis despite an insignificant result in univariate analysis. Cumulative cisplatin dose is frequently used as an endpoint of chemotherapy compliance. [14]–[16] However, the prognostic effect was not established. One study showed that survival was insignificantly improved with a cumulative cisplatin dose of 200 mg/m^2^. [17] This interesting finding will immediately raise a question in our mind. Did the result biased by the patients who did not complete the adjuvant treatment? In the current study, results was not changed after removing the patient who did not complete radiotherapy. On the other hand, CCRT did not correlate with incomplete radiotherapy as we mentioned in results. Therefore, cumulative cisplatin dose was an important prognostic factor.

Unlike the pooled analysis of 2 randomized trials, which suggested that the presence of multiple-node metastases is not an indication for concurrent chemotherapy, [3] this study suggests that concurrent chemotherapy can provide some benefit for this group of patients. There are several issues that need to be explored to explain the difference in the results. First, the cancer type in this study was solely OSCC, whereas OSCCs made up only approximately 25% of cancers in those randomized trials. Tumors in different locations usually have different outcomes and failure patterns. OSCC was considered a poor prognostic factor in some studies. [18], [19] Thus, more intensive treatment may be required for OSCC with the same pathologic findings. Second, the current study found that the benefit became significant after the total dose of cisplatin reached a lower limit. Dose of chemotherapy was reduced in 20% of the patients in CCRT arm of those 2 randomized trials. However, the correlation between compliance of chemotherapy and treatment outcome was not analyzed since those trials were not designed to answer this questions. The last reason to explain the difference in result is that most of patients in the current study had other risk factors of tumor recurrence. A study showed that coexistence of 3 or more risk factors of tumor recurrence in OSCC, tumor recurrence rate was high and adjuvant CCRT should be considered. The last issue is that multiple nodal metastases without extracapsular spreading is uncommon. Patients of the current study comprised less than 5% of the whole OSCC cancer registry. A study that is not specifically to the minority patients may not be able to reveal the benefit of different treatment modality.

In this study, concurrent chemotherapy was the only way to reduce the risk of tumor recurrence and improve survival. Other treatment factors did not correlate with outcome. In a literature review, dose escalation through conventional fractionation was effective only for patients with extracapsular spreading, and concurrent chemotherapy was not applied. [18] In the current study, a higher radiation dose (66 Gy) did not improve RFS or local-regional control. Another study also showed that a higher radiation dose does not produce a better result for oral tongue squamous cell carcinoma in a PORT setting [13]. It appears that concurrent chemotherapy is more effective than or masks the advantage of dose escalation in advanced OSCC patients. Time factors, such as an elapsed time between surgery and PORT of more than 6 weeks and a duration of PORT of more than 8 weeks, did not result in inferior OS or RFS. In most studies, time factors play a critical role in tumor control. Delays in starting postoperative treatment and prolonged treatment times were found to be correlated with more local-regional recurrence in a systematic review, a retrospective analysis, and clinical trials. [18], [20], [21]. The reasonable explanation for this result is that the risk of recurrence was lower than the risk of extracapsular spreading or positive resection margin. However, there is no need to investigate the issue of time factors. Because initiating and completing PORT in a limited time range would not cause more complications, delays in the initiation or completion of PORT should be avoided. The radiotherapy technique, that is, the conventional technique with center dose calculation, 3DCRT, or IMRT, did not alter the treatment outcome. Although the use of IMRT or 3DCRT resulted in better outcomes with respect to OS and RFS, these associations were not significant in either the univariate or multivariate analysis. Despite there being no advantages in tumor control, IMRT is inarguably better able to prevent complications and improve quality of life. [19], [22], [23] Therefore, IMRT is still the first technique of choice for PORT.

As the result of this study, patients will not acquire the benefit of concurrent chemotherapy if adequate dose of cisplatin cannot be given. Unfortunately, there is no reliable method to predict the compliance of cisplatin-based chemotherapy. Do we have an option other than cisplatin-based chemotherapy to improve the treatment outcome? The mortality rate during PORT was 2.2% in the current study, which is comparable to the mortality rates found in the randomized trials. [1], [2] However, if there is any less toxic but equally effective regimen that can be used in place of cisplatin, that regimen should be chosen. Primary radiotherapy plus cetuximab can reduce local-regional recurrence and mortality without increasing toxic effects during the treatment of local-regional advanced head and neck cancer. [24] Primary radiotherapy plus cetuximab is an attractive choice for the treatment of head and neck cancer. Unfortunately, there are no published results from prospective clinical trials using cetuximab with radiotherapy for the definitive or postoperative treatment of OSCC. One retrospective study reported that cetuximab is inferior to cisplatin in tumor control and survival in head and neck cancer patients. [25] Therefore, replacing cisplatin with cetuximab should be proven to be safe in a clinical trial before use in the clinic. Cisplatin remains our suggestion for concurrent chemoradiation. But if poor compliance of cisplatin-based chemotherapy can be predicted, radiotherapy plus cetuximab can be considered since inadequate cisplatin dose is ineffective.

## Conclusion

For OSCC patients with multiple-node metastases without extracapsular spreading or positive resection margins, PORT with concurrent chemotherapy is likely to decrease the risk of tumor recurrence, especially when cumulative cisplatin dose reached 200 mg/m^2^. Multiple lymph node metastases might be considered an optional indication for concurrent chemotherapy. Factors associated with cisplatin tolerance should be studied to improve the management for OSCC patients. Further prospective studies are required to confirm these results.
